# Aldosterone Is Positively Associated With Circulating FGF23 Levels in Chronic Kidney Disease Across Four Species, and May Drive FGF23 Secretion Directly

**DOI:** 10.3389/fphys.2021.649921

**Published:** 2021-04-29

**Authors:** Judith Radloff, Maximilian Pagitz, Olena Andrukhova, Rainer Oberbauer, Iwan A. Burgener, Reinhold G. Erben

**Affiliations:** ^1^Department of Biomedical Sciences, University of Veterinary Medicine Vienna, Vienna, Austria; ^2^Division of Small Animal Internal Medicine, Department for Companion Animals and Horses, University of Veterinary Medicine Vienna, Vienna, Austria; ^3^Department of Nephrology, Medical University of Vienna, Vienna, Austria

**Keywords:** fibroblast growth factor-23, chronic kidney disease, aldosterone, left ventricular hypertrophy, dialysis, calcimimetics

## Abstract

**Background:**

Chronic kidney disease (CKD) is accompanied by increases in circulating fibroblast growth factor 23 (FGF23) and aldosterone levels. Here, we tested the hypothesis that aldosterone may be one of the driving forces behind increased FGF23 secretion in CKD.

**Methods:**

Using data from a prospective study in humans, a retrospective study in dogs and cats, and an experimental study in 5/6-nephrectomized mice, we analyzed the relationship between circulating FGF23 and serum aldosterone levels in CKD across four species. To assess the effects of acute inhibition of aldosterone signaling on circulating FGF23, we acutely treated mice with established CKD with the mineralocorticoid receptor blocker canrenone (50 mg/kg iv/sc), and measured intact FGF23 before and 24 h as well as 72 h after start of administration of the drug.

**Results:**

We found a tight positive association between circulating intact FGF23 and serum aldosterone in human, canine, and feline CKD patients, as well as in experimental murine CKD (humans: *r*_S_ = 0.57, *p* = 0.0368; dogs: *r*_S_ = 0.66, *p* = 0.0019; cats: *r*_S_ = 0.75, *p* = 0.0003; mice: *r*_S_ = 0.49, *p* = 0.0004). Injection of canrenone in mice with established CKD did not lead to changes in FGF23 levels within 24 h, but reduced FGF23 in all mice at 72 h.

**Conclusion:**

Aldosterone may drive enhanced FGF23 secretion in CKD, possibly explaining the tight positive association between circulating intact FGF23 and aldosterone in human, canine, and feline CKD patients as well as in experimental CKD models.

## Introduction

Aldosterone is the body’s main mineralocorticoid hormone. Its production in the adrenal gland is triggered by potassium, angiotensin II, and, to a lesser extent, adrenocorticotropin (Hattangady et al., 2012). As part of the renin-angiotensin-aldosterone system (RAAS), aldosterone mainly promotes sodium reabsorption in the kidney, resulting in volume retention (Hattangady et al., 2012). At the same time, aldosterone augments urinary excretion of potassium. Serum levels of aldosterone are elevated in chronic kidney disease (CKD), contributing to volume overload and accelerated disease progression due to the development of hypertension (Greene et al., 1996; Hostetter and Ibrahim, 2003). In accordance with an important pathophysiological role of aldosterone in CKD, selective inhibition of aldosterone signaling reduced glomerular sclerosis, lowered arterial blood pressure, and lessened cardiac hypertrophy in 5/6-nephrectomized rats (Greene et al., 1996). In humans, inhibition of the RAAS system reduces mortality in patients with heart failure and CKD (Pitt et al., 1999; Kim et al., 2019). However, several studies have shown that despite RAAS blockade and improved clinical symptoms, circulating aldosterone levels continue to rise after an initial drop in approximately 40% of all patients (MacFadyen et al., 1999; Schjoedt et al., 2004). This aldosterone escape could be a contributing factor in the progression of CKD, because there is evidence that aldosterone may also play a direct role in the development of renal injury and proteinuria (Nagase et al., 2006).

Fibroblast growth factor 23 (FGF23) is a 32 kD proteo-hormone mainly secreted from bone cells, reducing phosphate reabsorption from urine by the downregulation of sodium phosphate co-transporters in proximal renal tubular epithelium (Shimada et al., 2001, 2004, 2005; Andrukhova et al., 2012). In addition, FGF23 downregulates 1α-hydroxylase expression in proximal renal tubules, thereby suppressing the production of the biologically active vitamin D hormone, 1α,25-dihydroxyvitamin D_3_ [1,25(OH)_2_D_3_] (Shimada et al., 2001, 2004). In typical FGF23 target tissues such as the kidney, the biological effects of FGF23 are mediated through a receptor complex consisting of FGF receptors (FGFRs) and of the co-receptor αKlotho (Urakawa et al., 2006; Chen et al., 2018). FGF23 may also be a modulator of RAAS. It has been shown that FGF23 is involved in the activation of local RAAS in the heart of rats and humans, promoting cardiac hypertrophy and fibrosis (Leifheit-Nestler et al., 2018; Böckmann et al., 2019). It is currently unknown whether FGF23 directly modulates RAAS in the kidney.

Reciprocally to declining renal function, FGF23 levels continue to rise stage-dependently in CKD patients. In advanced CKD stages, circulating intact FGF23 can be increased 1,000-fold above normal (Gutierrez et al., 2005). Elevated FGF23 levels increase the risk of mortality and disease progression to end-stage renal disease in human CKD patients, making it a clinically relevant biomarker (Isakova et al., 2011). Similarly, in canine and feline CKD patients, circulating intact FGF23 rises stage-dependently (Geddes et al., 2013; Harjes et al., 2017). A retrospective study in 214 cats with CKD revealed that survival over 12 months was negatively associated with circulating intact FGF23 (Geddes et al., 2015). Furthermore, circulating intact FGF23 predicted the development of CKD over a 12-month follow-up period in a prospective study in healthy aged cats (Geddes et al., 2013). Taken together, these data suggest that circulating intact FGF23 is also associated with adverse outcomes in feline and canine CKD patients similar to humans (Geddes et al., 2013, 2015; Harjes et al., 2017).

The mechanisms driving bony FGF23 secretion in CKD are currently unclear. Because extracellular phosphate stimulates FGF23 secretion from bone *in vivo* (Erben, 2017), it was previously believed that hyperphosphatemia triggers the augmented skeletal FGF23 secretion in CKD as part of an adaptive physiological response (Erben, 2017; Harjes et al., 2017). However, later studies in both humans and cats have shown that the increase in circulating intact FGF23 occurs independently of hyperphosphatemia and increased parathyroid hormone (PTH) in early CKD stages (Shimamura et al., 2012; Mace et al., 2015), suggesting that other stimuli must be involved in the upregulation of FGF23 secretion in CKD patients. In this context, it is conceivable that reduced renal elimination of FGF23 due to declining kidney function (Mace et al., 2015), renal FGF23 resistance due to a CKD-associated downregulation of renal αKlotho expression (Shimamura et al., 2012), or increased secretion of FGF23-stimulating substances such as aldosterone or pro-inflammatory cytokines may be involved in the upregulation of blood concentrations of intact FGF23 in CKD (David et al., 2016; Erben, 2016; Kovesdy and Quarles, 2016; Zhang et al., 2016). It was recently shown that aldosterone augments FGF23 expression in cultured osteoblast-like cells *in vitro* (Zhang et al., 2016). Hence, it is tempting to speculate that increased circulating aldosterone might be a key driver of enhanced FGF23 secretion in CKD patients.

In the current study, we examined the relationship between circulating FGF23 and serum aldosterone levels in CKD patients and in an experimental CKD model, using data from a prospective study in humans, a retrospective study in cats and dogs, and an experimental study in 5/6-nephrectomized (5/6-Nx) mice. In addition, we tested the hypothesis that aldosterone may be a direct driving factor of increased FGF23 secretion in CKD in a mouse study.

## Materials and Methods

### Study in Human CKD Patients

The human study was approved by the Ethical Committee of the Medical University of Vienna on November 17, 2016 (#2006/2016), and each patient provided written consent for the use of the serum for this study. We prospectively collected serum from 16 patients with various stages of chronic kidney disease (CKD stage 2–5), who underwent a diagnostic kidney biopsy. Each patient received a full set of routine lab work including electrolytes, creatinine, aldosterone, and intact FGF23 (ELISA, Kainos) measurements. Overall, 16 Caucasian study participants were included in the analysis (Table 1). Both genders were equally represented (8 each). The median age of the participants was 57 years, ranging from 20 to 78 years. Patients were categorized according to estimated glomerular filtration rate (eGFR in ml/min/1.73 m^2^) into 5 different groups: G2 (eGFR = 60–89; *n* = 5), G3a (eGFR = 45–59; *n* = 1), G3b (eGFR = 30–44; *n* = 3), G4 (eGFR = 15–29; *n* = 4), and G5 (eGFR < 15; *n* = 3). Renal injury in most patients was caused by glomerulonephritis of different causes (systemic Lupus erythematodes = 2, IgA glomerulonephritis = 8). Renal Damage in the rest of the patients was caused by atypical hemolytic-uremic syndrome (1), diabetic glomerulosclerosis (1), p-ANCA associated systemic vasculitis (1), cysts (1), and amyloidosis (1).

**TABLE 1:** Demographic and health parameters of the human patient population.

Gender and Age	eGFR	Cause of renal injury
Female (*n* = 8) Median age: 55 Age range: 27–78	G2 (2)	Glomerulonephritis, aHus
	G3b (1)	p-ANCA associated vasculitis
	G4 (2)	Glomerulonephritis
	G5 (3)	Glomerulonephritis, Amyloidosis
Male (*n* = 8) Median age: 58 Age range: 20–72	G2 (3)	Glomerulonpehritis
	G3a (1)	Glomerulonephritis
	G3b (2)	Diabetic glomerulosclerosis, renal cysts
	G4 (2)	Glomerulonpehritis, unknown

### Study in Canine and Feline CKD Patients

The retrospective study in cats and dogs was approved by the Institutional Ethics and Animal Welfare Committee of the University of Veterinary Medicine Vienna, and all owners signed a general consent form allowing the later use of non-used samples. Client-owned dogs and cats with or without CKD were retrospectively recruited from the patient population referred to the Teaching Hospital of the University of Veterinary Medicine Vienna. Unused blood samples after routine blood sampling of dogs and cats were collected and stored at –80°C. Serum samples of eligible patients fulfilling the inclusion criteria of the study were later analyzed for intact FGF23 and aldosterone. The diagnosis of CKD IRIS (International Renal Interest Society) stage ≥ 2 was made based on the presence of a history of polyuria and polydipsia, azotemia, and urine specific gravity < 1.030 in dogs and urine specific gravity < 1.035 in cats over at least 3 months. Dogs and cats were assigned to IRIS stages 2–4 for CKD based on plasma creatinine concentrations (dogs: 1.4–2.0 mg/dL—stage 2; 2.1–5.0 mg/dL—stage 3; and > 5.0 mg/dL— stage 4; cats: 1.6–2.8 mg/dL—stage 2; 2.9–5.0 mg/dL—stage 3; and > 5.0 mg/dL— stage 4). Dogs and cats presented with non-renal diseases without impact on parathyroid function, and those with diseases with possible impact on renal function, but normal plasma creatinine values were assigned to the IRIS stage 0/1 group. All animals in IRIS stage 0/1 had plasma creatinine and urine specific gravity in the normal range. Dogs and cats < 1 year of age, and those diagnosed with acute kidney injury, primary parathyroid diseases, suspected recent acute-to-chronic kidney disease transition, and animals receiving corticosteroids or angiotensin-converting enzyme (ACE) inhibitors were excluded from the study. Overall, 1 intact male, 22 neutered males, 21 neutered female cats and 11 intact male, 5 neutered male, 11 intact female, and 8 neutered female dogs were included in this study.

### Experimental Murine CKD Model

The experimental mouse study was approved by the Animal Welfare Committee of the Austrian Federal Ministry of Science and Research and was undertaken in strict accordance with the guidelines for animal care (permit No. BMWF–68.205/0054-II/3b/2013). All experiments were performed in 14–16-weeks old male C57Bl/6 mice. Mice were kept at 24°C with a 12/12-h light/dark cycle and had *ad libitum* access to food and water. CKD was surgically induced via 5/6-Nx under isoflurane anesthesia as previously described (Andrukhova et al., 2018). Buprenorphine and metamizole were applied as analgesic drugs before surgery. Postsurgical pain management involved application of metamizole for 3 days, as well as buprenorphine for at least 1 day. In the first surgery, 2/3 of the left kidney were removed. Seven days later, mice underwent total nephrectomy for their right kidney. Because it is well known that C57Bl/6 mice are relatively resistant to the development of CKD after 5/6-Nx, mice were fed a diet containing 2.0% calcium, 1.25% phosphorus, and 20% lactose (Ssniff, Soest, Germany), which accelerates renal injury due to the high phosphate content (Lau et al., 2013). 5/6-Nx C57Bl/6 mice on this phosphate-enriched diet show an about 50–60% reduction in glomerular filtration rate as assessed by creatinine clearance 8 weeks postsurgery (Xu et al., 2019). In Sham mice left and right kidneys were exposed and repositioned before closing the flank incision. Mice were exsanguinated from the abdominal Vena cava under general anaesthesia (ketamine/xylazine, 100/6 mg/kg i.p.) for serum collection 4, 8, or 12 weeks postsurgery. Samples were stored at –80°C.

To test the putative FGF23-stimulating role of aldosterone signaling in this disease model, 5/6-Nx mice with established CKD (12 weeks postsurgery) received a single iv injection at 0 h for the 24 h time point or two sc injections at 0 and 48 h for the 72 h time point with the aldosterone receptor blocker canrenone (50 mg/kg, Soldactone, Pfizer, Switzerland). Blood for measurement of circulating intact FGF23 was taken from the submandibular vein immediately before and 24 or 72 h after start of canrenone treatment.

### Clinical Chemistry

Serum creatinine was measured on a Hitachi 911 autoanalyzer (Roche), and on a Cobas c111 analyzer (Roche) in dogs, cats and mice, respectively. Serum intact FGF23 in all species was analyzed using an immunoassay detecting intact FGF23 across many species (Kainos, Tokyo, Japan). This assay has previously also been validated in cats and dogs (Geddes et al., 2013; Harjes et al., 2017). Serum intact aldosterone was determined by ELISA (NovaTec Immundiagnostica, Dietzenbach, Germany) after extraction of the serum using diethyl ether, and resuspension in equal amounts of steroid-free human serum (DRG).

### Statistical Analyses

Statistics were computed using Prism 8.4 (GraphPad Software Inc., La Jolla, CA, United States). The data were analyzed by Spearman’s rank correlation analysis to reduce the effects of outliers, followed by linear regression analysis. Serum levels of FGF23 before and after treatment with canrenone were compared using Wilcoxon test. *P* < 0.05 were considered significant.

## Results and Discussion

**FIGURE 1:**
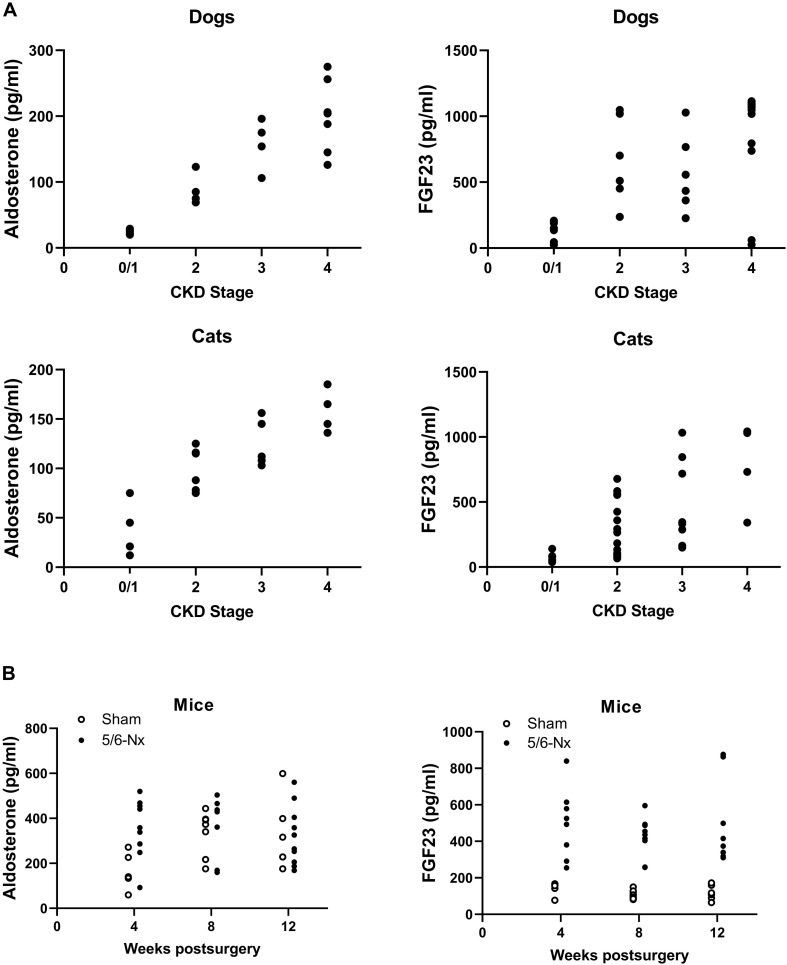
Stage-dependent increase in aldosterone and circulating FGF23 in clinical and experimental CKD. **(A)**, Stage-dependent increase in aldosterone and circulating intact FGF23 in cats and dogs with CKD. **(B)**, Aldosterone and intact FGF23 serum levels in Sham and 5/6-Nx mice as an experimental CKD model, 4, 8, and 12 weeks postsurgery.

**FIGURE 2:**
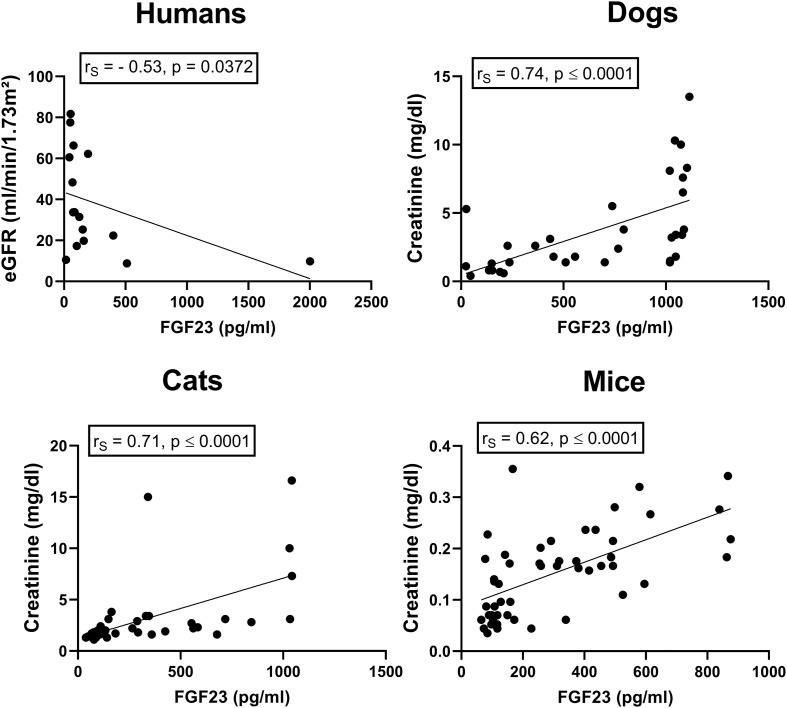
Association between renal function and circulating intact FGF23 in human, feline, and canine CKD patients as well as in experimental murine CKD. Intact serum FGF23 and estimated glomerular filtration rate (eGFR) as well as serum creatinine levels are correlated in human (*n* = 16), canine (*n* = 34), and feline (*n* = 38) CKD patients as well as in mice with experimentally induced CKD (*n* = 50). Insets show Spearman rank correlation coefficients.

In accordance with previous studies and the well-known fact that CKD is associated with activation of the RAAS (Hostetter and Ibrahim, 2003), we found a stage-dependent increase in circulating intact FGF23 and aldosterone in dogs and cats with CKD (Figure 1A). Similarly, circulating intact FGF23 and aldosterone rose with increasing time postsurgery in experimental CKD in 5/6-nephrectomized mice maintained on a phosphate-enriched diet (Figure 1B). Serum intact FGF23 was closely associated with eGFR in human CKD patients (humans: *r*_S_ = –0.53, *p* = 0.0372), as well as with plasma creatinine in canine and feline CKD patients and in 5/6-Nx mice (dogs: *r*_S_ = 0.74, *p* < 0.0001; cats: *rs* = 0.71, *p* < 0.0001; mice: *r*_S_ = 0.62, *p* < 0.0001; Figure 2). These results confirm the tight association between elevated serum intact FGF23 and declining renal function across all four species tested.

**FIGURE 3:**
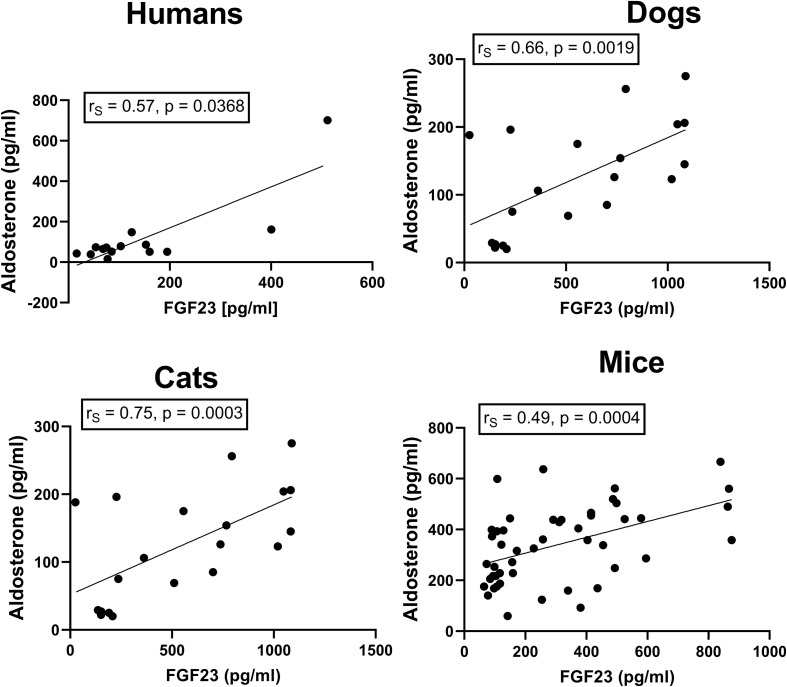
Association between circulating FGF23 with serum aldosterone in human, feline, and canine CKD patients as well as in experimental murine CKD. Serum intact FGF23 and aldosterone levels are positively correlated in humans (*n* = 14), dogs (*n* = 20), and cats (*n* = 18) with CKD, as well as in mice with experimentally induced CKD (*n* = 48). Insets show Spearman rank correlation coefficients.

**FIGURE 4:**
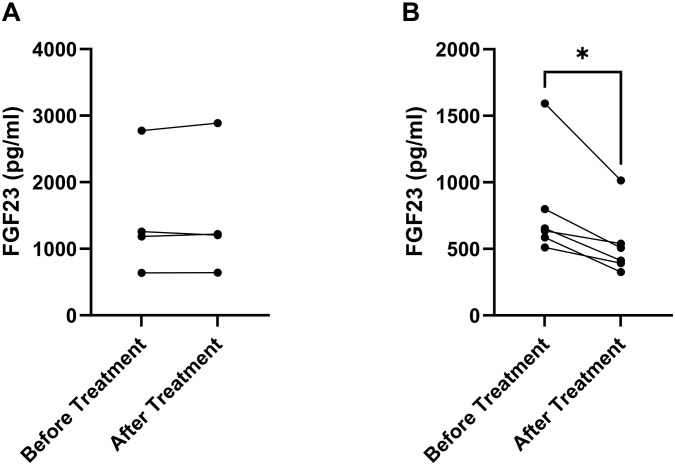
Acute inhibition of aldosterone signaling lowers circulating FGF23 in mice with CKD. Serum levels of circulating intact FGF23 before and 24 h **(A)** or 72 h **(B)** after a single iv injection **(A)** or two sc injections at 0 and 48 h **(B)** of the mineralocorticoid receptor antagonist canrenone to mice with established CKD, 12 weeks after 5/6-Nx (*n* = 4–6 each). *P < 0.05 by Wilcoxon test.

The factors driving FGF23 secretion in CKD are still controversial. One of the candidates may be aldosterone, which has been shown to stimulate FGF23 expression in cultured osteoblast-like cells *in vitro* (Zhang et al., 2016). When we analyzed the association between serum aldosterone concentrations and circulating intact FGF23, we found strikingly close correlations across all four species (humans: *r*_S_ = 0.57, *p* = 0.0368; dogs: *r*_S_ = 0.66, *p* = 0.0019; cats: *r*_S_ = 0.75, *p* = 0.0003; mice: *r*_S_ = 0.49, *p* = 0.0004; Figure 3). A recent cross-sectional study in 180 human non-dialysis CKD patients stages 1–5 also found a positive correlation between serum aldosterone and circulating intact FGF23 (Xu et al., 2019). These findings would be in line with the notion that the elevated aldosterone levels in CKD patients may directly drive FGF23 secretion in bone. To test this hypothesis, we administered a high dose (50 mg/kg) of the mineralocorticoid receptor blocker canrenone to mice with established CKD, 12 weeks post-5/6-Nx. Canrenone is the active metabolite produced from the pro-drug spironolactone (Yang and Young, 2016). To assess the effects of inhibition of the aldosterone signaling pathway on circulating FGF23, we measured the blood concentrations of intact FGF23 in each animal immediately before and 24 h after iv injection of canrenone, or after a 3-day treatment with canrenone injected sc at time 0 and 48 h. Circulating FGF23 remained uninfluenced by acute inhibition of aldosterone signaling, 24 h post-injection (Figure 4A). However, 3 days of aldosterone inhibition downregulated serum intact FGF23 in all treated mice on average by ∼30% (Figure 4B). These data suggest that aldosterone is a direct driver of FGF23 secretion in 5/6-nephrectomized mice, but that the effect of aldosterone inhibition is evident only after several days of treatment.

It is of course conceivable that the tight association between serum aldosterone concentrations and circulating intact FGF23 in humans, dogs, cats and mice with CKD is not only based on a direct stimulating effect of aldosterone on FGF23 secretion but may have additional components. Both parameters rise with renal disease progression. Therefore, there may be indirect links associated with progressing renal disease such as a rise in pro-inflammatory cytokines. In addition, it has been reported that angiotensin II stimulates osteoblastic FGF23 secretion (Kovesdy and Quarles, 2016). Hence, it is possible that the stimulating effect of the RAAS on FGF23 occurs also at the level of angiotensin II, and not only at the level of aldosterone. Further experiments are required to test this scenario. Moreover, it is well known that FGF23 is a powerful suppressor of renal 1,25(OH)_2_D_3_ production. Although we were not able to reproduce this finding (Andrukhova et al., 2014), Li et al. (2002) reported that 1,25(OH)_2_D_3_ suppresses renin gene expression. Therefore, the 1,25(OH)_2_D_3_ lowering effect of FGF23 might indirectly stimulate renin expression in the kidney. To the best of our knowledge, short-term intervention studies examining the effects of RAAS inhibition on circulating FGF23 in CKD patients do not exist. Therapeutic use of RAAS inhibitors to lower blood pressure and to prevent further loss of renal function is common in CKD (Pitt et al., 1999; Kim et al., 2019), but potential disease progression-independent effects of RAAS inhibitors on circulating FGF23 are unclear. In addition, the use of angiotensin or aldosterone blockers in CKD patients can also lead to a compensatory increase in serum renin levels, and, therefore, unwanted RAAS activation (Cully, 2013), which in turn could even aggravate disease progression.

Another indirect link between RAAS and FGF23 may be a modulating effect of RAAS activation on renal FGF23 resistance. Mitani and coworkers reported that activated RAAS, represented by angiotensin II, downregulated the FGF23 co-receptor αKlotho in experimental animal models (Mitani et al., 2002). Renal Klotho expression decreases in the early stages of CKD, which may lead to a compensatory increase of circulating FGF23 due to renal FGF23 resistance, because FGF23 needs αKlotho to bind to FGF receptors (Urakawa et al., 2006; Shimamura et al., 2012). In agreement with a potential role of the RAAS on Klotho expression in CKD, the application of ACE inhibitors in a rat model of diabetic nephropathy restored αKlotho expression and attenuated FGF23 upregulation, also limiting renal injury (Zanchi et al., 2013). Therefore, a putative direct suppressive effect of RAAS activation on renal αKlotho expression in CKD could be one of the factors leading to upregulation of FGF23 production in the bone.

In conclusion, we found a tight positive association between circulating intact FGF23 and aldosterone in human, canine, and feline CKD patients as well as in experimental murine CKD. In accordance with this finding, a 3-day treatment of 5/6-Nx mice with the aldosterone receptor blocker canrenone distinctly downregulated the elevated serum FGF23 levels, suggesting that aldosterone is one of the drivers of FGF23 secretion in CKD. Because the 3-day treatment of 5/6-Nx mice with canrenone lowered serum intact FGF23 only by about 30%, it is likely that other factors also contribute to enhanced FGF23 secretion in CKD. It is clear that more work needs to be done to draw the full picture of the mechanism(s) underlying enhanced FGF23 secretion in CKD.

## Data Availability Statement

The original contributions presented in the study are included in the article/supplementary material, further inquiries can be directed to the corresponding author/s.

## Ethics Statement

The studies involving human participants were reviewed and approved by the Ethical Committee of the Medical University of Vienna. The patients/participants provided their written informed consent to participate in this study. The animal study was reviewed and approved by the Ethics and Animal Welfare Committee of the University of Veterinary Medicine Vienna. Written informed consent was obtained from the owners for the participation of their animals in this study.

## Author Contributions

JR, MP, OA, RO, IB, and RE conceived and designed the studies. JR, MP, OA, and RE performed the experiments, collected the samples, and analyzed the data. JR, MP, IB, and RE wrote the manuscript. All authors discussed and reviewed the manuscript.

## Conflict of Interest

The authors declare that the research was conducted in the absence of any commercial or financial relationships that could be construed as a potential conflict of interest.
